# Thymic T-Cell Production Is Associated With Changes in the Gut Microbiota in Young Chicks

**DOI:** 10.3389/fimmu.2021.700603

**Published:** 2021-09-10

**Authors:** Jiaheng Cheng, Yushan Yuan, Fang Zhao, Jianwei Chen, Peng Chen, Ying Li, Xia Yan, Chenglong Luo, Dingming Shu, Hao Qu, Jian Ji

**Affiliations:** ^1^State Key Laboratory of Livestock and Poultry Breeding, Guangdong Key Laboratory of Animal Breeding and Nutrition, Institute of Animal Science, Guangdong Academy of Agricultural Sciences, Guangzhou, China; ^2^BGI-Qingdao, BGI-Shenzhen, Qingdao, China; ^3^Qingdao-Europe Advanced Institute for Life Sciences, BGI-Shenzhen, Qingdao, China

**Keywords:** gut microbiota, T cell, thymus, antibiotic, early life

## Abstract

Increasing studies show that gut microbiota play a central role in immunity, although the impact of the microbiota on mediation of thymic T cells throughout life is not well understood. Chickens have been shown to be a valuable model for studying basic immunology. Here, we show that changes in the gut microbiota are associated with the development of thymic T cells in young chickens. Our results showed that T-cell numbers in newborn chicks sharply increased from day 0 and peaked at day 49. Interestingly, the α-diversity score pattern of change in gut microbiota also increased after day 0 and continued to increase until day 49. We found that early antibiotic treatment resulted in a dramatic reduction in gut alpha diversity: principal component analysis (PCA) showed that antibiotic treatment resulted in a different cluster from the controls on days 9 and 49. In the antibiotic-treated chickens, we identified eight significantly different (*p* < 0.05) microbes at the phylum level and 14 significantly different (*p* < 0.05) microbes at the genus level, compared with the controls. Importantly, we found that antibiotic treatment led to a decreased percentage and number of T cells in the thymus when measured at days 9 and 49, as evaluated by flow cytometry. Collectively, our data suggest that intestinal microbiota may be involved in the regulation of T cells in birds, presenting the possibility that interventions that actively modify the gut microbiota in early life may accelerate the maturation of humoral immunity, with resulting anti-inflammatory effects against different pathogens.

## Introduction

T cells, a type of lymphocyte, are central to the adaptive immune response (1). T cells are derived from hematopoietic stem cells in the bone marrow, migrate to the thymus for differentiation and maturation, and are then transported to the periphery to carry out immune functions (2). In early life, T cells can remove pathogens and generate memory responses (3), and also establish tolerance to harmless antigens (4).

Gut microbiota consist of complex mixtures of microorganisms that have coevolved over time, building symbiotic relationships with their hosts (5). The gut microbiota is known to play a significant role in the development of the immune system, organismal health, and disease (6–9), and changes in gut microbiota in early life can profoundly impact the host immune system (10). For example, the gut microbiota of preterm infants differs in composition from that of full-term infants. Preterm infants also display a different developmental trajectory of their peripheral immune cell populations, compared with full-term infants (11). Interference with the early gut microbiota leads to impaired thymocyte development in mice, and these effects persist into adulthood (12). In addition, alteration of microbiota structure in early life as a consequence of antibiotic treatment in piglets can lead to excessive inflammation, local tissue damage, and a possible increased risk of immune-mediated disease following pathogenic bacterial infection (13). In mouse models, antibiotic-treated and germ-free mice show distinct intestinal T-cell receptor (TCR) repertoires compared with their wild-type controls, suggesting that microbial antigens alter T-cell development (14). Interestingly, T cells were first found in the thymus of chicken (15). Therefore, the chicken offered an excellent animal model in which to further study the relationship between the host immunity and gut microbiota.

The mechanism(s) by which thymic T cells may be affected by gut microbiota at different stages of growth, however, is poorly understood. Here, we provide further evidence that gut microbiota play a critical role in the early development of T cells in chickens.

## Materials and Methods

### Animal Study and Experimental Design

Chinese Yellow broiler breeders (Lingnan) used in this study were obtained from a local hatchery (Lingnan, Guangdong Wiz Agricultural Science & Technology Co. Ltd., Guangzhou, China) and were cared for and used in accordance with the guidelines of Guangdong Province on the Review of Welfare and Ethics of Laboratory Animals and approved by the Guangdong Province Administration Office of Laboratory Animals. The birds were fed a corn and soybean meal-based diet, formulated to meet their nutritional requirements.

A total of 80 one-day-old broilers were randomly divided into two equal groups and fed the same diet with the antibiotic-treatment group receiving drinking water containing penicillin (200 mg/L), metronidazole (200 mg/L), and vancomycin (100 mg/L) (16). Cecal samples were collected from chickens on days 0 (freshly hatched chickens were not given antibiotic), 9, 49, and 140. When birds were sacrificed by approved methods, each thymus was immediately prepared for histological and flow cytometric investigations. Cecal contents were collected and stored at −80°C for further analysis.

### Histology

Each thymus was collected, and portions were fixed in PBS containing 10% neutral-buffered formalin. Paraffin-embedded sections (5 μm) were stained with hematoxylin and eosin.

### Flow Cytometric Analysis

Fresh thymus was collected, minced with scissors, and filtered through a 100-μm nylon cell strainer (BD Falcon, San Jose, CA, USA). The cells were suspended in PBS after washing. Total cell numbers were determined using a hemocytometer. Suspended cells were centrifuged at 1,200 rpm for 5 min at 4°C. The single-cell suspension was stained with fluorochrome-conjugated mouse antichicken T antibody (Cat. No. 8200-31, SouthernBiotech, Birmingham, AL, USA) for 30 min on ice. After staining, cells were washed and analyzed using a FACS Calibur flow cytometer (Becton Dickinson, Palo Alto, CA, USA) and Cell Quest software (Becton Dickinson).

### Microbial Genomic DNA Extraction

The microbial composition of cecal contents from birds was determined from microbial genomic DNA, as previously described (17).

### Bacterial 16S rDNA Gene Sequencing

The primer sequences for amplification of the V4 region were as follows: forward 5′-GTGCCAGCMGCCGCGGTAA-3′ and reverse 5′-GGACTACHVGGGTWTCTAAT-3′. The melting temperature was 55°C, and 25 PCR cycles were run. The validated libraries were subjected to paired-end sequencing using the MGISEQ-2000 platform.

### Sequencing Data Analysis

Raw sequence reads were preprocessed to eliminate adapter pollution and low quality to obtain clean reads. Paired-end clean reads with overlaps were merged to tags by Fast Length Adjustment of Short reads (FLASH, v1.2.11) (18). Bacterial tags were clustered into operational taxonomic units (OTU) at 97% sequence identity using USEARCH (v7.0.1090) (19). OTU taxonomic classification was conducted by scripts of QIIME (v1.8.0) software based on the Ribosomal Database Project (RDP) database (20). Observed species and Shannon’s index were calculated using Mothur (v1.31.2), and the rarefaction curves were drawn using R (v3.4.1) software. Beta diversity analysis based on weighted UniFrac distance was conducted by QIIME (v1.80) software. The resulting distance matrices were visualized using PCA with the “vegan” package of R (v3.4.1).

### Statistical Analysis

Statistical analyses were performed using the GraphPad Prism 8.0 Software (La Jolla, CA, USA). Comparisons between treatment and controls were performed using the Student’s *t*-test. *p*-Values <0.05 were considered to be statistically significant. Taxa abundances at the phylum and genus levels, and the alpha diversity comparisons between two groups, were made by the Wilcoxon test and visualized through box-and-whisker plots. The significance of differences in microbiota composition between groups was assessed by permutational multivariate analysis of variance (PERMANOVA) using the R package “vegan”.

## Results

### Changes in Thymic Structure in Chickens Following Antibiotic Treatment

**Figure 1:**
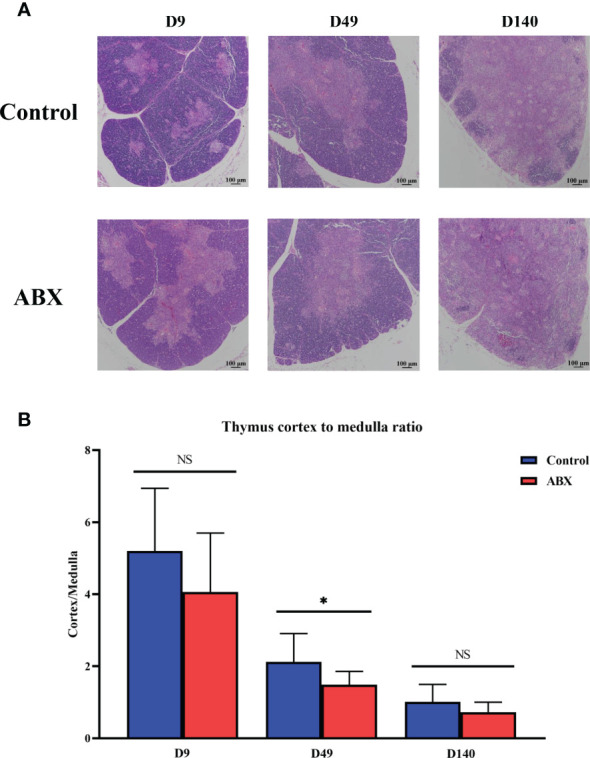
Antibiotic treatment leads to structural changes in the thymic tissue. **(A)** Light micrograph of the thymus stained with hematoxylin and eosin at different ages. Magnification, ×100; scale bar, 100 μm. **(B)** Differences in the ratio of thymic cortex to medulla at different ages in the controls (Ctr) and antibiotic-treated chickens (ABX). *p < 0.05; unpaired *t*-test. *n* ≥ 10 for each group. ns, not significant.

The thymus is composed of two histologically discrete regions—an outer region known as the cortex, where a stromal meshwork houses densely packed immature thymocytes, and an inner region known as the medulla, which is the region with less densely localized mature thymocytes. Our results showed that the corticomedullary ratio of the thymus gland tended to decrease in the antibiotic-treated group compared with the controls on day 9 (*p* > 0.05). On day 49, the antibiotic-treated chickens had a significantly greater decrease in the corticomedullary ratio of the thymus gland than the controls (*p* < 0.05), with a further ratio decrease on day 140 (**Figures 1A, B** and **Figure S1**). Collectively, these data suggest that antibiotic-induced thymus dysbiosis may have significant long-term effects on chicken T cells.

### Effects of Antibiotic Treatment on the Proportion and Number of Thymic T Cells

**Figure 2:**
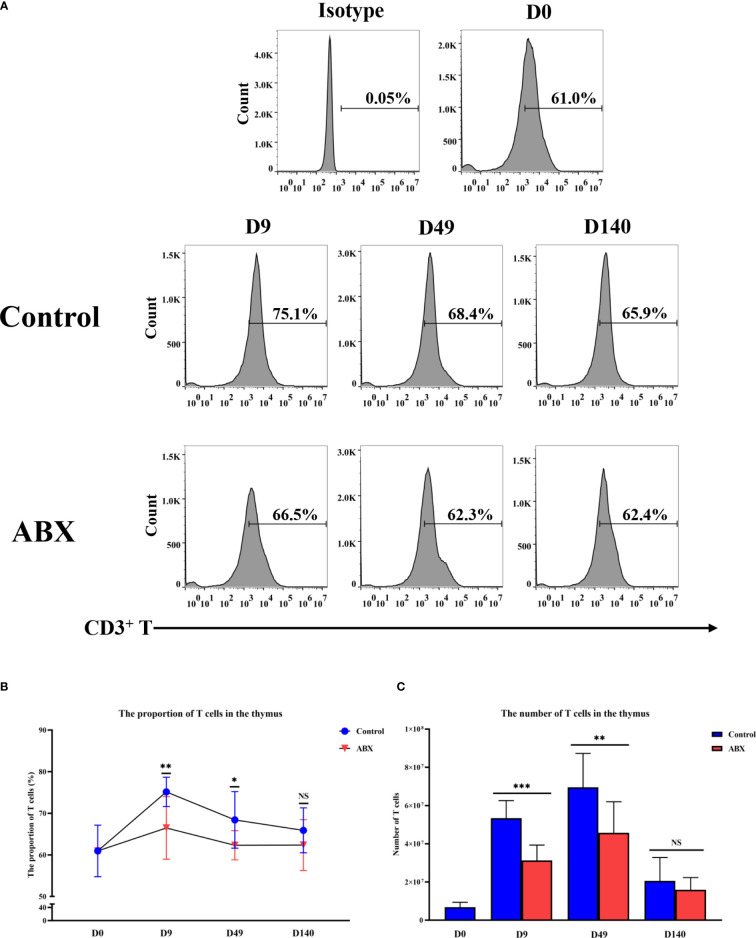
Flow cytometric analysis of T-cell numbers and proportions in thymic tissue. **(A)** Detection of T-cell content in thymic tissue at different ages by flow cytometry. **(B)** The proportion of T cells in thymic tissue. **(C)** The number of T cells in thymic tissue. *p < 0.05, **p < 0.01, ***p < 0.001; unpaired *t*-test. Ctr, controls; ABX, antibiotic-treated chickens. *n* ≥ 12 for each group. ns, not significant.

Flow cytometry was used to quantify the proportion and numbers of T cells in the thymus at different ages (days 0, 9, 49, and 140). On days 9 and 49, both proportion and number of CD3^+^ T cells in the antibiotic-treated group were higher than in the control group (*p* < 0.05), while no significant difference was observed on day 140 (*p* > 0.05) (**Figures 2A–C**). These results suggest that the potential influence of gut microbiota on thymic T cells may be much stronger in younger chickens.

### Effects of Antibiotic Treatment on the Diversity of the Gut Microbiota

**Figure 3:**
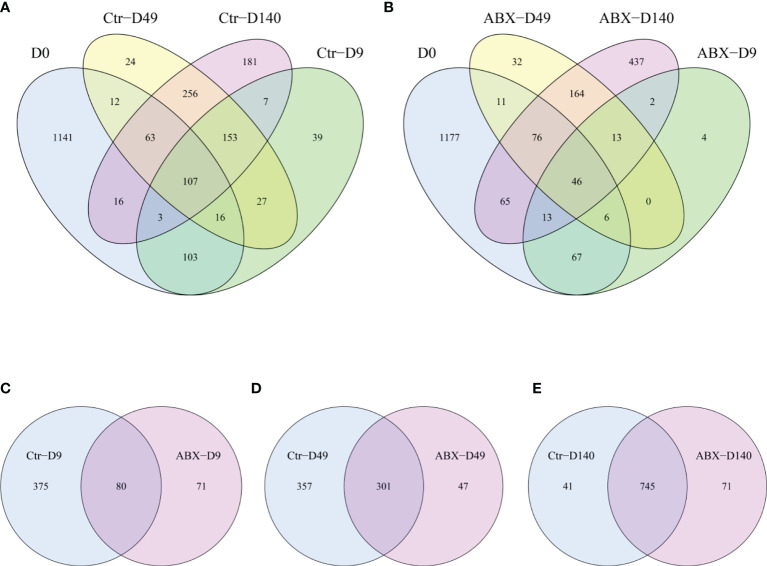
Overall structural changes in gut microbiota with antibiotic treatment. **(A)** Differences in the distribution of operational taxonomic units (OTU) at different ages in the controls (Ctr) were analyzed using Venn diagrams. **(B)** Differences in the distribution of OTU at different ages in birds treated with antibiotics (ABX) were analyzed using Venn diagrams. **(C)** Differences in OUT between Ctr and ABX on day 9 of antibiotic treatment, **(D)** on day 49 of antibiotic treatment, and **(E)** day 140. *n* ≥ 8 for each group.

To better investigate the role of gut microbiota in chicken thymic T cells, 16s rDNA gene sequencing was used to characterize changes in the overall structure of the gut microbiota at different ages (days 0, 9, 49, and 140). **Figures 3A, B** shows that the core microbiota at different life stages (days 0, 9, 49, and 140) comprised 107 OTU in controls, but only 46 OUT in birds treated with antibiotic. We found that 80, 301, and 745 OTU were shared by controls and chicks treated with antibiotics on days 9, 49 and 140, respectively (**Figures 3C–E**). Compared with the controls, the total number of OUT decreased in the antibiotic-treated birds on days 9 and 49 (**Figures 3C–E**).

**Figure 4:**
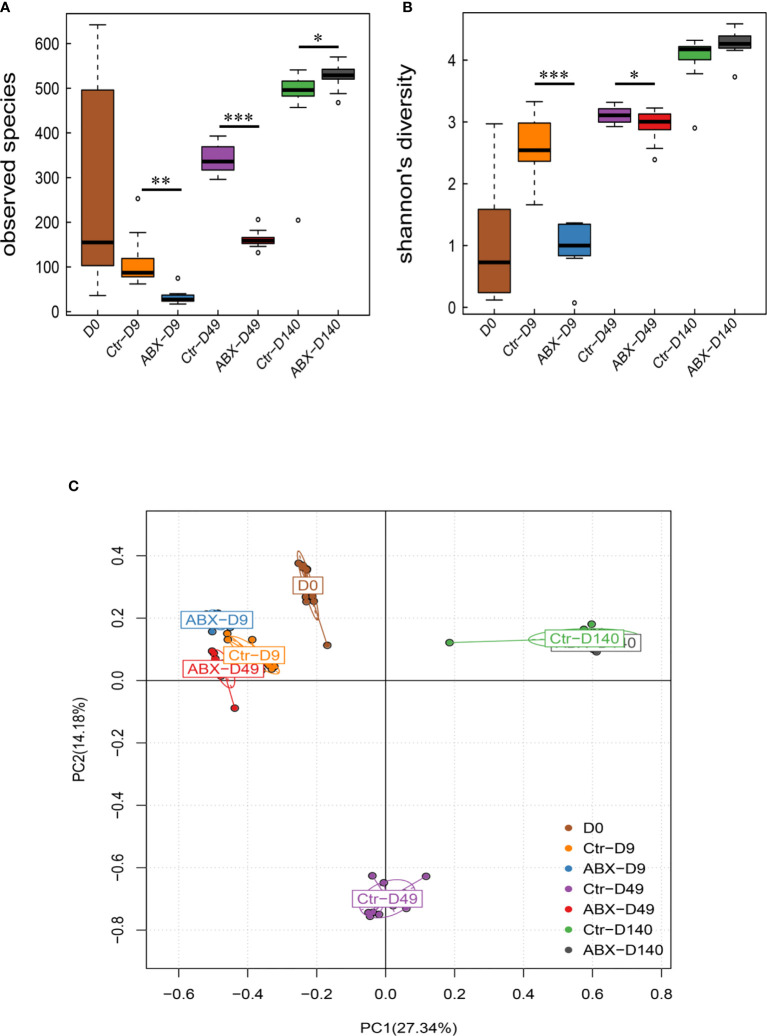
Effects of antibiotic treatment on the diversity of the gut microbiota. **(A)** Differences in α-diversity between control (Ctr) and antibiotic-treated (ABX) chickens at different ages. **(B)** Differences in α-diversity between Ctr and ABX at different ages analyzed with the Shannon’s indices. **(C)** Differences in β-diversity between Ctr and ABX groups at different ages analyzed with principal component analysis. *p < 0.05, **p < 0.01, ***p < 0.001; Wilcoxon rank-sum test. *n* ≥ 8 for each group.

We next studied the changes in the alpha diversity of the gut microbiota at different days in antibiotic-treated chicks. There were significant differences in observed species between the antibiotic-treated birds and the controls at the three ages (days 9, 49, and 140) (*p* < 0.05). Shannon’s indices showed that the diversity was lower in the antibiotic-treated chickens than in the controls on days 9 and 49 (*p* < 0.05) (**Figures 4A, B** **)**. We additionally assessed intersample variability in the community structure (beta diversity) using an unsupervised principal component analysis (PCA) of Bray-Curtis dissimilarity. The PCA clearly showed statistical differences between the chickens treated with antibiotic and controls, particularly on day 49 (*p* < 0.05); however, no clear clusters were present for either group on day 140 (**Figure 4C**). These data suggest that changes in the gut microbiota were also related to chicken age, and that antibiotic exposure during critical periods of early development may significantly influence gut microbiota in chickens.

### Antibiotic-Induced Alterations of Gut Microbiome Populations at the Phylum and Genus Levels

**Figure 5:**
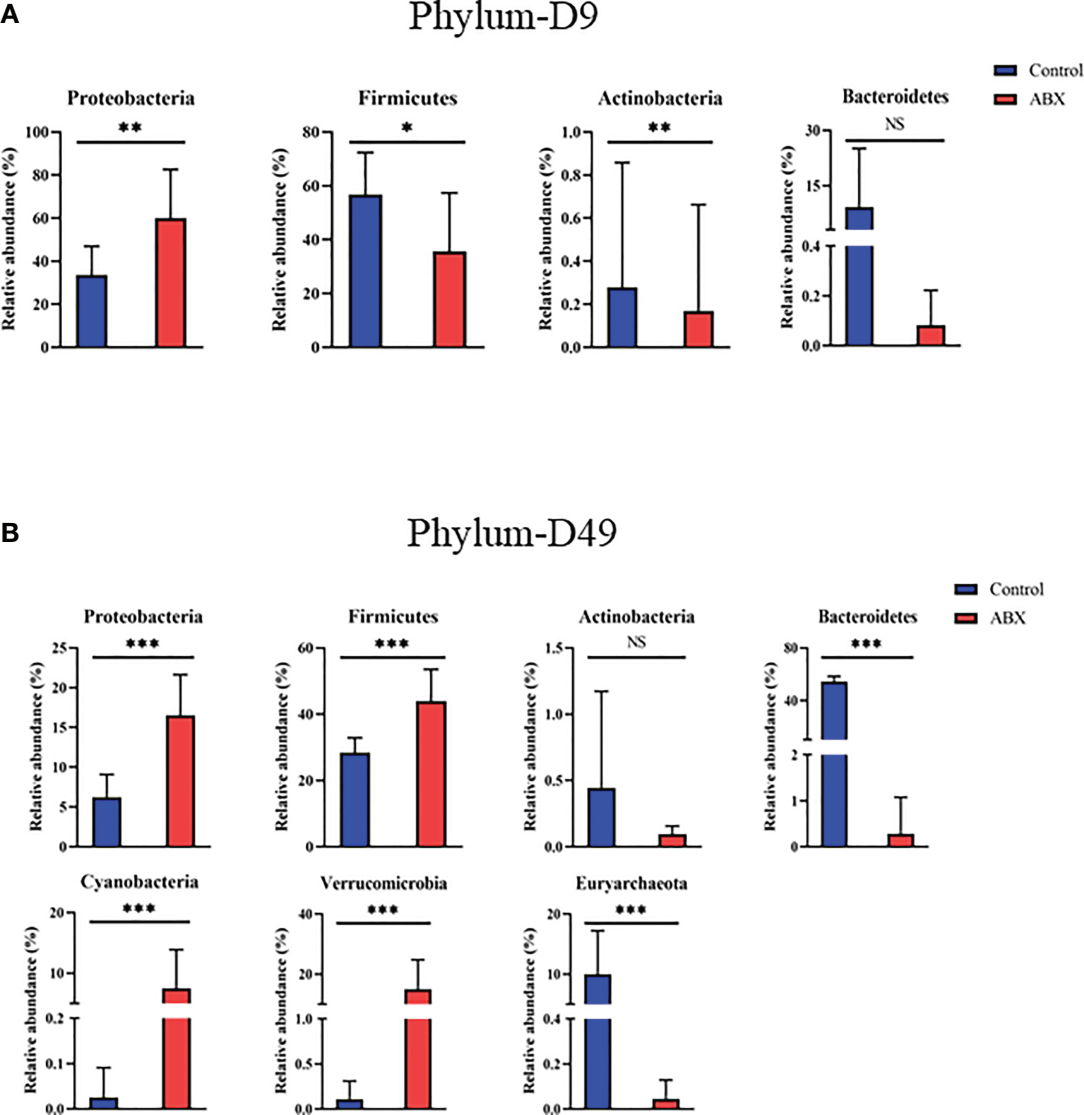
Relative abundance of the gut microbiota at the phylum level showing significant changes in response to antibiotic treatment during early life. **(A)** Differences in relative abundance of phyla between the controls (Ctr) and antibiotic treatment (ABX) on day 9. **(B)** Differences in relative abundance of genera between Ctr and ABX on day 49. *p < 0.05, **p < 0.01, ***p < 0.001; Wilcoxon rank-sum test. *n* ≥ 8 for each group. ns, not significant.

To further understand changes in microbial community composition, we compared the relative abundance of different bacteria in the antibiotic-treated and control chickens at the bacterial phylum and genus levels. The chicken gut microbiota included hundreds of bacterial species dominated at the phylum level by *Firmicutes*, *Bacteroidetes*, *Proteobacteria*, and *Actinobacteria*. Dominant at the genus level were *Escherichia*, *Sutterella*, *Ruminococcus*, *Parabacteroides*, *Oscillospira*, *Clostridium*, *Bacteroides*, *Eubacterium*, and *Blautia*. At the phylum level, 25 phyla were used (**Figure S2A**); of these, four phyla differed significantly (*p* < 0.05) between the controls and antibiotic-treated chickens on day 9 (**Figure 5** **)**. With antibiotic treatment, relative abundances of *Firmicutes* and *Bacteroidetes* were significantly decreased (*p* < 0.05), whereas *Proteobacteria* was significantly increased (*p* < 0.05) (**Figure 5A**). On day 49, we observed a decrease in *Bacteroidetes* and *Euryarchaeota* and an increase in *Proteobacteria*, *Verrucomicrobia*, *Cyanobacteria*, and *Firmicutes* in the antibiotic-treated *versus* control chickens (*p* < 0.05) (**Figure 5B**). On day 140, there was no significant difference between the control and antibiotic-treated birds (*p* > 0.05) (data not shown).

**Figure 6:**
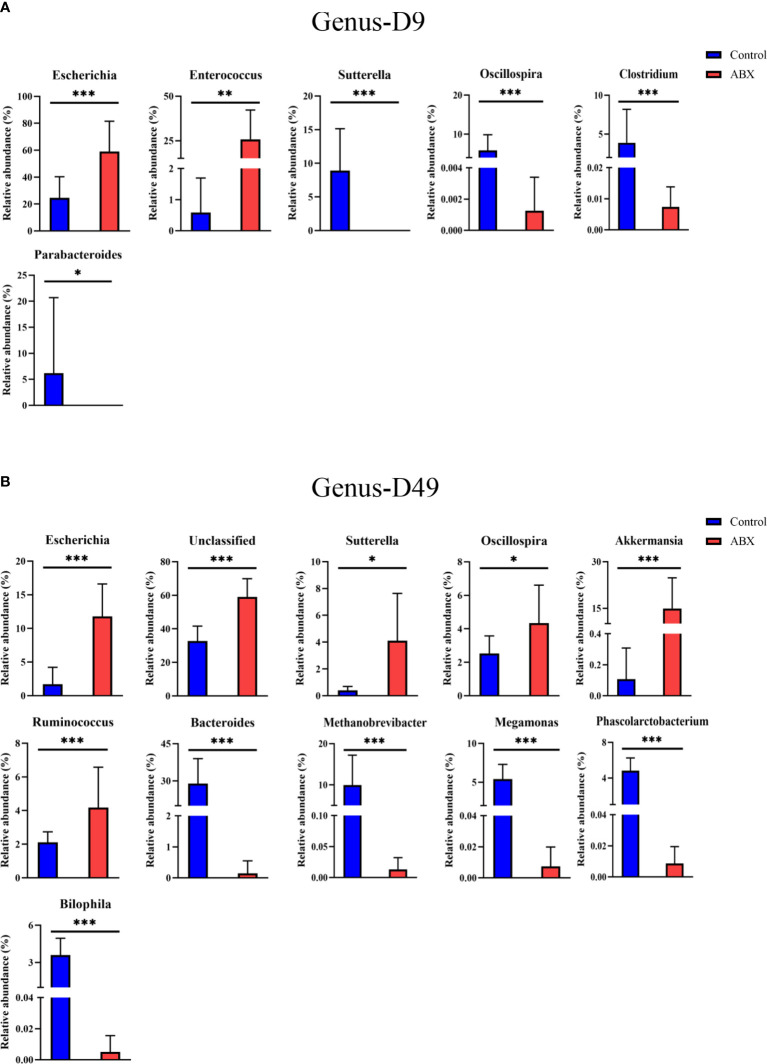
Gut microbiota showing significant changes in relative abundance at the phylum and genus level in response to antibiotic treatment during early life. **(A)** Differences between the controls (Ctr) and antibiotic-treated birds (ABX) in phyla on day 9. **(B)** Differences in genera. *p < 0.05, **p < 0.01, ***p < 0.001; Wilcoxon rank-sum test. *n* ≥ 8 for each group.

At the genus level, 105 genera were used (**Figure S2B**). On day 9, in the antibiotic-treated chickens, *Escherichia* and *Enterococcus* showed a relatively higher abundance (*p* < 0.05) compared with the controls, while *Sutterella*, *Oscillospira*, *Clostridium*, and *Parabacteroides* showed a lower relative abundance (*p* < 0.05) (**Figure 6A**). On day 49, the relative abundances of *Bacteroides*, *Methanobrevibacter*, *Megamonas*, *Phascolarctobacterium*, and *Bilophila* were significantly decreased in the antibiotic-treated group compared with the control group (*p* < 0.05), while the relative abundances of *Escherichia*, *Sutterella*, *Oscillospira*, *Akkermansia*, and *Rumnococcus* were significantly increased (*p* < 0.05) (**Figure 6B**). On day 140, only one genus, *Methanobrevibater*, showed different patterns of distribution in the control and antibiotic groups (*p* < 0.05) (**Figure S3**). These results further show the effects of antibiotic treatment on gut microbial composition, especially at early ages in the life cycle. As these changes are correlated with changes in thymic structure in chickens following antibiotic treatment, as described above, the results further suggest that gut microbiota potentially influence thymic T cells, especially early in life.

## Discussion

Gut microbes are known to have a profound impact on the development of the immune system (21–23). In the present study, we attempted to provide a comprehensive, time-dimensional analysis of the association between gut microbes and thymic T-cell development in birds.

The thymus has two main cellular zones including the cortex, and the smaller central zone, the medulla, which is essential for the production and generation of T cells (24–27). Here, we found that in chickens, the antibiotic-treated chickens had a significantly greater decrease in the cortex-medullary ratio of the thymus gland than the controls at early ages. These structural changes may be associated with T-cell development in birds. To determine whether the composition of gut microbiota is reflected in the development of thymic T cells, we measured the numbers and proportions of thymic T cells by flow cytometry, with and without antibiotic treatment of the birds. The administration of broad-spectrum antibiotics reduced the proportion and number of the thymic T cells compared with the controls on days 49 and 140. Previous studies have shown that vancomycin treatment leads to a reduction of the number of Tregs and impairs the induction of Th17 cells in the colonic lamina propria in mice (28).

Research on the effect of gut microbiota on the immune system has provided increasing evidence that the gut microbiota is a critical factor in the differentiation and development of immune cells. Germ-free animals show extensive deficits in the development of the gut-associated lymphoid tissues and defects in antibody production (29–31). In our experiment, 16S rDNA gene sequencing showed that eight significantly different microbes were present at the phylum level in antibiotic-treated birds. Interestingly, in one example, the relative abundances of *Proteobacteria* significantly increased on days 9 and 49 with antibiotic treatment. *Proteobacteria*, a phylum that comprises several known human pathogens, is a potential diagnostic signature of dysbiosis and risk of disease. Stunted children also have altered gut bacterial communities with higher proportions of *Proteobacteria* (32, 33).

In addition, in younger chickens, we found that the relative abundance of *Bacteroidetes* was significantly decreased in the birds treated with antibiotics compared with the control group. It has been shown previously that the lack of *Bacteroidetes* inhibits T-cell differentiation (34). *Bacteroidetes* are crucial for early colonization of germfree mice, which then results in the correction of their underdeveloped immune system (35). At the genus level, antibiotic-treated *versus* control chickens showed 14 significant differences at days 9 and 49. One difference was the relative abundance of *Escherichia* in the antibiotic-treated birds compared with the controls on days 9 and 49. Interestingly, *Escherichia* has been reported to inhibit the proliferation and differentiation of T cells (36, 37). This result emphasized that more work should be performed to further explore the interaction between gut microbes and host thymic T cells. A recent study showed that intestinal microbial colonization in early life causes the trafficking of microbial antigens from the intestine to the thymus through intestinal dendritic cells, which then induce the expansion of microbiota-specific T cells in mice (38). It is important to discover whether similar thymic trafficking of gut microbiota that induces T cells occurs in birds, also driving the expansion of thymic T cells. For example, microbial metabolites SCFAs in gut and CD4^+^ T-cell subsets would be determined in the future because SCFAs can be transported into the blood and then to thymic tissue sites where they may have the potential to regulate T-cell activity.

Our results emphasize the need for more work to further explore the interaction between gut microbes and host thymic T cells. Future studies should clarify which gut microbiota may specifically stimulate T-cell development in the thymus, and such bacteria might be used as a feed additive for birds. A better understanding of the development of thymic T cells in early life, including how to open the development window and start thymic generation by the specified gut microbiota, may eventually provide new ways to treat immune disorders, such as inflammatory bowel disease.

## Conclusion

In summary, the results of the present study indicate that the spectrum of gut microbiota correlated with the percentage and number of thymic T cells early in the lives of these birds, and this model may be useful in understanding the role of the gut microbiota in modulating immunity.

## Data Availability Statement

The datasets presented in this study can be found in online repositories. The names of the repository/repositories and accession number(s) can be found in the article/**Supplementary Material**.

## Ethics Statement

All the birds used in this study were cared for and used in accordance with the guidelines of Guangdong Province on the Review of Welfare and Ethics of Laboratory Animals and approved by the Guangdong Province Administration Office of Laboratory Animals.

## Author Contributions

JJ and HQ conceived and designed the experiments. JJ, JCheng, YY, PC, XY, and YL performed the experiments. JJ, HQ, JCheng, JChen, FZ, DS, and CL analyzed the data. JJ wrote the paper. All authors contributed to the article and approved the submitted version.

## Funding

Financial support was provided from the Key Realm R&D Program of Guangdong Province (2020B0202090004), the Special fund for Scientific Innovation Strategy-Construction of High Level Academy of Agriculture Science (R2020PY-JX006, 202107TD), the Modern Agricultural Industrial Technology System of Guangdong Province (2020KJ128), and the China Agriculture Research System of MOF and MARA (CARS-41).

## Conflict of Interest

The authors declare that the research was conducted in the absence of any commercial or financial relationships that could be construed as a potential conflict of interest.

## Publisher’s Note

All claims expressed in this article are solely those of the authors and do not necessarily represent those of their affiliated organizations, or those of the publisher, the editors and the reviewers. Any product that may be evaluated in this article, or claim that may be made by its manufacturer, is not guaranteed or endorsed by the publisher.

## References

[1] Gaber T Chen Y Krauß PL Buttgereit F. Metabolism of T Lymphocytes in Health and Disease. Int Rev Cell Mol Biol (2019) 342:95–148. doi: 10.1016/bs.ircmb.2018.06.002 30635095

[2] Liu C Lan Y Liu B Zhang H Hu H. T Cell Development: Old Tales Retold By Single-Cell RNA Sequencing. Trends Immunol (2021) 42(2):165–75. doi: 10.1016/j.it.2020.12.004 33446417

[3] Kumar BV Connors TJ Farber DL. Human T Cell Development, Localization, and Function Throughout Life. Immun (2018) 48(2):202–13. doi: 10.1016/j.immuni.2018.01.007 PMC5826622 29466753

[4] Goronzy JJ Weyand CM. Successful and Maladaptive T Cell Aging. Immun (2017) 46(3):364–78. doi: 10.1016/j.immuni.2017.03.010 PMC5433436 28329703

[5] Caruso R Lo BC Núñez G. Host-Microbiota Interactions in Inflammatory Bowel Disease. Nat Rev Immunol (2020) 20(7):411–26. doi: 10.1038/s41577-019-0268-7 32005980

[6] Michaudel C Sokol H. The Gut Microbiota at the Service of Immunometabolism. Cell Metab (2020) 32(4):514–23. doi: 10.1016/j.cmet.2020.09.004 32946809

[7] Brown EM Kenny DJ Xavier RJ. Gut Microbiota Regulation of T Cells During Inflammation and Autoimmunity. Annu Rev Immunol (2019) 37:599–624. doi: 10.1146/annurev-immunol-042718-041841 31026411

[8] Kim M Qie Y Park J Kim CH. Gut Microbial Metabolites Fuel Host Antibody Responses. Cell Host Microbe (2016) 20(2):202–14. doi: 10.1016/j.chom.2016.07.001 PMC4982788 27476413

[9] Honda K Littman DR. The Microbiota in Adaptive Immune Homeostasis and Disease. Nat (2016) 535(7610):75–84. doi: 10.1038/nature18848 27383982

[10] Gensollen T Iyer SS Kasper DL Blumberg RS. How Colonization by Microbiota in Early Life Shapes the Immune System. Sci (2016) 352(6285):539–44. doi: 10.1126/scienceaad9378 PMC5050524 27126036

[11] Olin A Henckel E Chen Y Lakshmikanth T Pou C Mikes J. Stereotypic Immune System Development in Newborn Children. Cell (2018) 174(5):1277–1292.e14. doi: 10.1016/j.cell.2018.06.045 30142345 PMC6108833

[12] Ennamorati M Vasudevan C Clerkin K Halvorsen S Verma S Ibrahim S. Intestinal Microbes Influence Development of Thymic Lymphocytes in Early Life. Proc Natl Acad Sci USA (2020) 117(5):2570–8. doi: 10.1073/pnas.1915047117 PMC7007548 31964813

[13] Fouhse JM Yang K More-Bayona J Gao Y Goruk S Plastow G. Neonatal Exposure to Amoxicillin Alters Long-Term Immune Response Despite Transient Effects on Gut-Microbiota in Piglets. Front Immunol (2019) 10:2059. doi: 10.3389/fimmu.2019.02059 31552023 PMC6737505

[14] Cebula A Seweryn M Rempala GA Pabla SS McIndoe RA Denning TL. Thymus-Derived Regulatory T Cells Contribute to Tolerance to Commensal Microbiota. Nat (2013) 497(7448):258–62. doi: 10.1038/nature12079 PMC3711137 23624374

[15] Cooper MD Peterson RD Good RA. Delineation of the Thymic and Bursal Lymphoid Systems in the Chicken. Nat (1965) 205:143–6. doi: 10.1038/205143a0 14276257

[16] Simon K Verwoolde MB Zhang J Smidt H de Vries Reilingh G Kemp B. Long-Term Effects of Early Life Microbiota Disturbance on Adaptive Immunity in Laying Hens. Poult Sci (2016) 95(7):1543–54. doi: 10.3382/ps/pew088 26976906

[17] Ji J Luo CL Zou X Lv XH Xu YB Shu DM. Association of Host Genetics With Intestinal Microbial Relevant to Body Weight in a Chicken F2 Resource Population. Poult Sci (2019) 98(9):4084–93. doi: 10.3382/ps/pez199 31330021

[18] Magoč T Salzberg SL. FLASH: Fast Length Adjustment of Short Reads to Improve Genome Assemblies. Bioinformatics (2011) 27(21):2957–63. doi: 10.1093/bioinformatics/btr507 PMC3198573 21903629

[19] Edgar RC. UPARSE: Highly Accurate OTU Sequences From Microbial Amplicon Reads. Nat Methods (2013) 10(10):996–8. doi: 10.1038/nmeth.2604 23955772

[20] Cole JR Wang Q Fish JA Chai B McGarrell DM Sun Y. Ribosomal Database Project: Data and Tools for High Throughput rRNA Analysis. Nucleic Acids Res (2014) 42(Database issue):D633–42. doi: 10.1093/nar/gkt1244 PMC3965039 24288368

[21] McCoy KD Burkhard R Geuking MB. The Microbiome and Immune Memory Formation. Immunol Cell Biol (2019) 97(7):625–35. doi: 10.1111/imcb.12273 31127637

[22] Yu Q Jia A Li Y Bi Y Liu G. Microbiota Regulate the Development and Function of the Immune Cells. Int Rev Immunol (2018) 37(2):79–89. doi: 10.1080/08830185.2018.1429428 29425062

[23] Fung TC Olson CA Hsiao EY. Interactions Between the Microbiota, Immune and Nervous Systems in Health and Disease. Nat Neurosci (2017) 20(2):145–55. doi: 10.1038/nn.4476 PMC6960010 28092661

[24] Kadouri N Nevo S Goldfarb Y Abramson J. Thymic Epithelial Cell Heterogeneity: TEC by TEC. Nat Rev Immunol (2020) 20(4):239–53. doi: 10.1038/s41577-019-0238-0 31804611

[25] Abramson J Anderson G. Thymic Epithelial Cells. Annu Rev Immunol (2017) 35:85–118. doi: 10.1146/annurev-immunol-051116-052320 28226225

[26] Swann JB Nusser A Morimoto R Nagakubo D Boehm T. Retracing the Evolutionary Emergence of Thymopoiesis. Sci Adv (2020) 6(48):eabd9585. doi: 10.1126/sciadv.abd9585 33246964 PMC7695478

[27] Alawam AS Anderson G Lucas B. Generation and Regeneration of Thymic Epithelial Cells. Front Immunol (2020) 11:858. doi: 10.3389/fimmu.2020.00858 32457758 PMC7221188

[28] Atarashi K Tanoue T Shima T Imaoka A Kuwahara T Momose Y. Induction of Colonic Regulatory T Cells by Indigenous Clostridium Species. Science (2011) 331(6015):337–41. doi: 10.1126/science.1198469 PMC3969237 21205640

[29] Nowosad CR Mesin L Castro T Wichmann C Donaldson GP Araki T. Tunable Dynamics of B Cell Selection in Gut Germinal Centres. Nat (2020) 588(7837):321–6. doi: 10.1038/s41586-020-2865-9 PMC7726069 33116306

[30] Li H Limenitakis JP Greiff V Yilmaz B Schären O Urbaniak C. Mucosal or Systemic Microbiota Exposures Shape the B Cell Repertoire. Nature (2020) 584(7820):274–8. doi: 10.1038/s41586-020-2564-6 32760003

[31] Legoux F Bellet D Daviaud C El Morr Y Darbois A Niort K. Microbial Metabolites Control the Thymic Development of Mucosal-Associated Invariant T Cells. Sci (2019) 366(6464):494–9. doi: 10.1126/science.aaw2719 31467190

[32] Khan Mirzaei M Khan M Ghosh P Taranu ZE Taguer M Ru J. Bacteriophages Isolated From Stunted Children Can Regulate Gut Bacterial Communities in an Age-Specific Manner. Cell Host Microbe (2020) 27(2):199–212.e5. doi: 10.1016/j.chom.2020.01.004 32053789 PMC7013830

[33] Shin NR Whon TW Bae JW. Proteobacteria: Microbial Signature of Dysbiosis in Gut Microbiota. Trends Biotechnol (2015) 33(9):496–503. doi: 10.1016/j.tibtech.2015.06.011 26210164

[34] Ivanov II Frutos R Manel N Yoshinaga K Rifkin DB Sartor RB. Specific Microbiota Direct the Differentiation of IL-17-Producing T-Helper Cells in the Mucosa of the Small Intestine. Cell Host Microbe (2008) 4(4):337–49. doi: 10.1016/j.chom.2008.09.009 PMC2597589 18854238

[35] Gibiino G Lopetuso LR Scaldaferri F Rizzatti G Binda C Gasbarrini A. Exploring Bacteroidetes: Metabolic Key Points and Immunological Tricks of Our Gut Commensals. Dig Liver Dis (2018) 50(7):635–9. doi: 10.1016/j.dld.2018.03.016 29650468

[36] Cassady-Cain RL Blackburn EA Bell CR Elshina E Hope JC Stevens MP. Inhibition of Antigen-Specific and Nonspecific Stimulation of Bovine T and B Cells by Lymphostatin From Attaching and Effacing *Escherichia Coli* . Infect Immun (2017) 85(2):e00845–16. doi: 10.1128/IAI.00845-16 PMC5278176 27920212

[37] Cassady-Cain RL Hope JC Stevens MP. Direct Manipulation of T Lymphocytes by Proteins of Gastrointestinal Bacterial Pathogens. Infect Immun (2018) 86(5):e00683–17. doi: 10.1128/IAI.00683-17 PMC5913853 29339462

[38] Zegarra-Ruiz DF Kim DV Norwood K Kim M Wu WH Saldana-Morales FB. Thymic Development of Gut-Microbiota-Specific T Cells. Nat (2021) 594(7863):413–7. doi: 10.1038/s41586-021-03531-1 PMC8323488 33981034

