# Supplementary figures and images for: Thymic T-Cell Production Is Associated With Changes in the Gut Microbiota in Young Chicks
Source: Front Immunol. 2021 Sep 10;12:700603. doi: 10.3389/fimmu.2021.700603 (PMC8461177; doi:10.3389/fimmu.2021.700603)

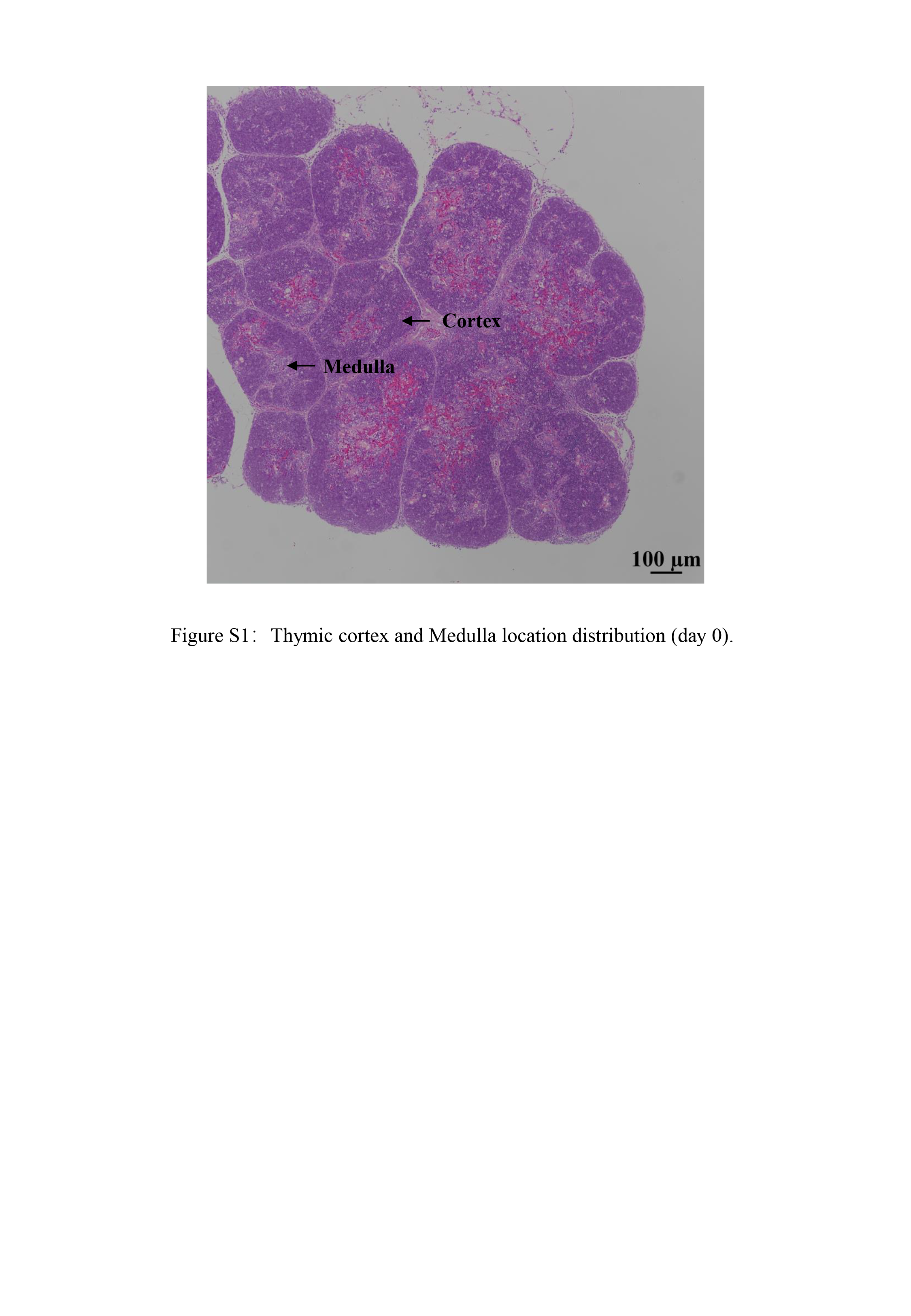

Supplement: Supplementary file 1 [file Image_1.tif]

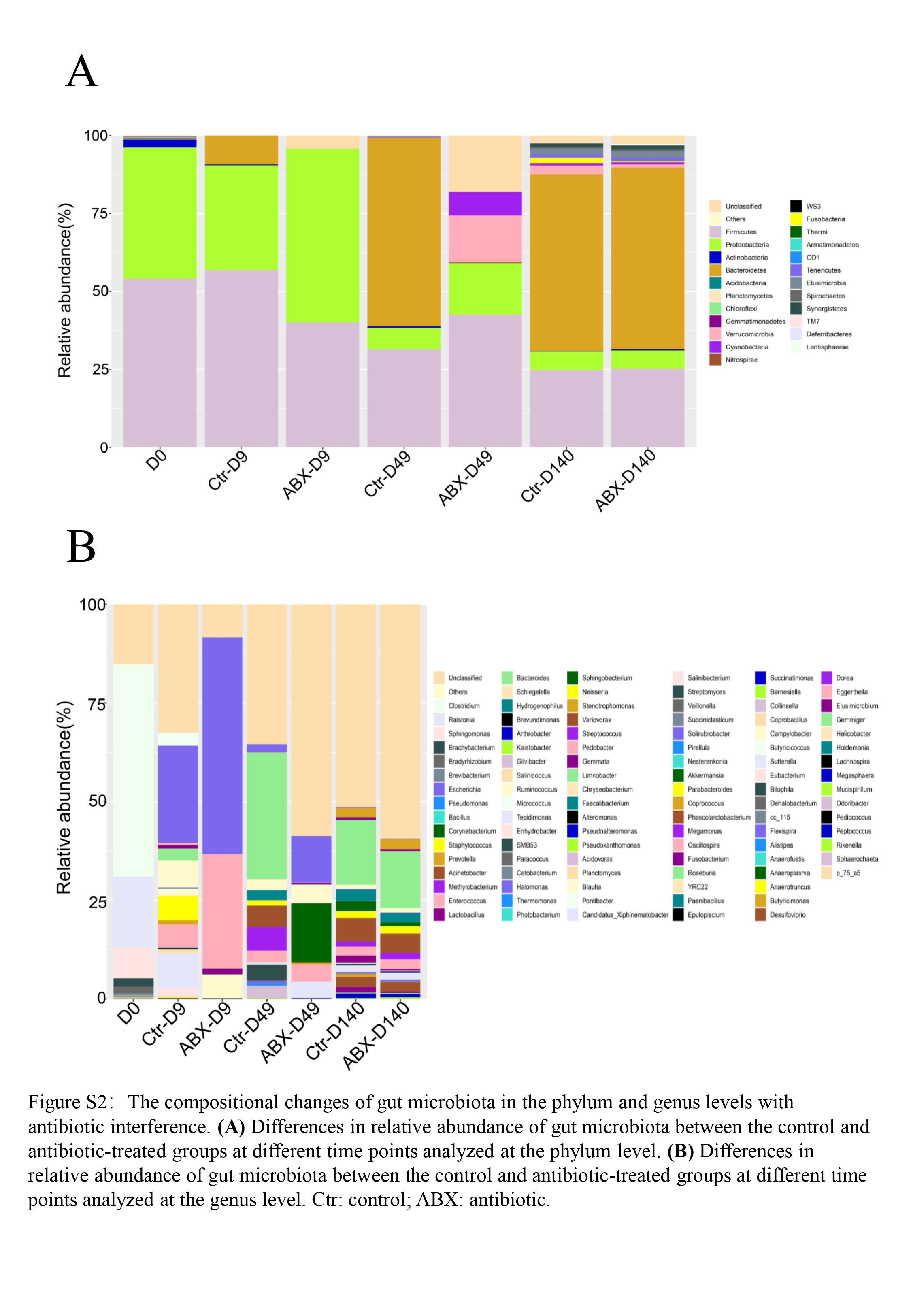

Supplement: Supplementary file 2 [file Image_2.tif]

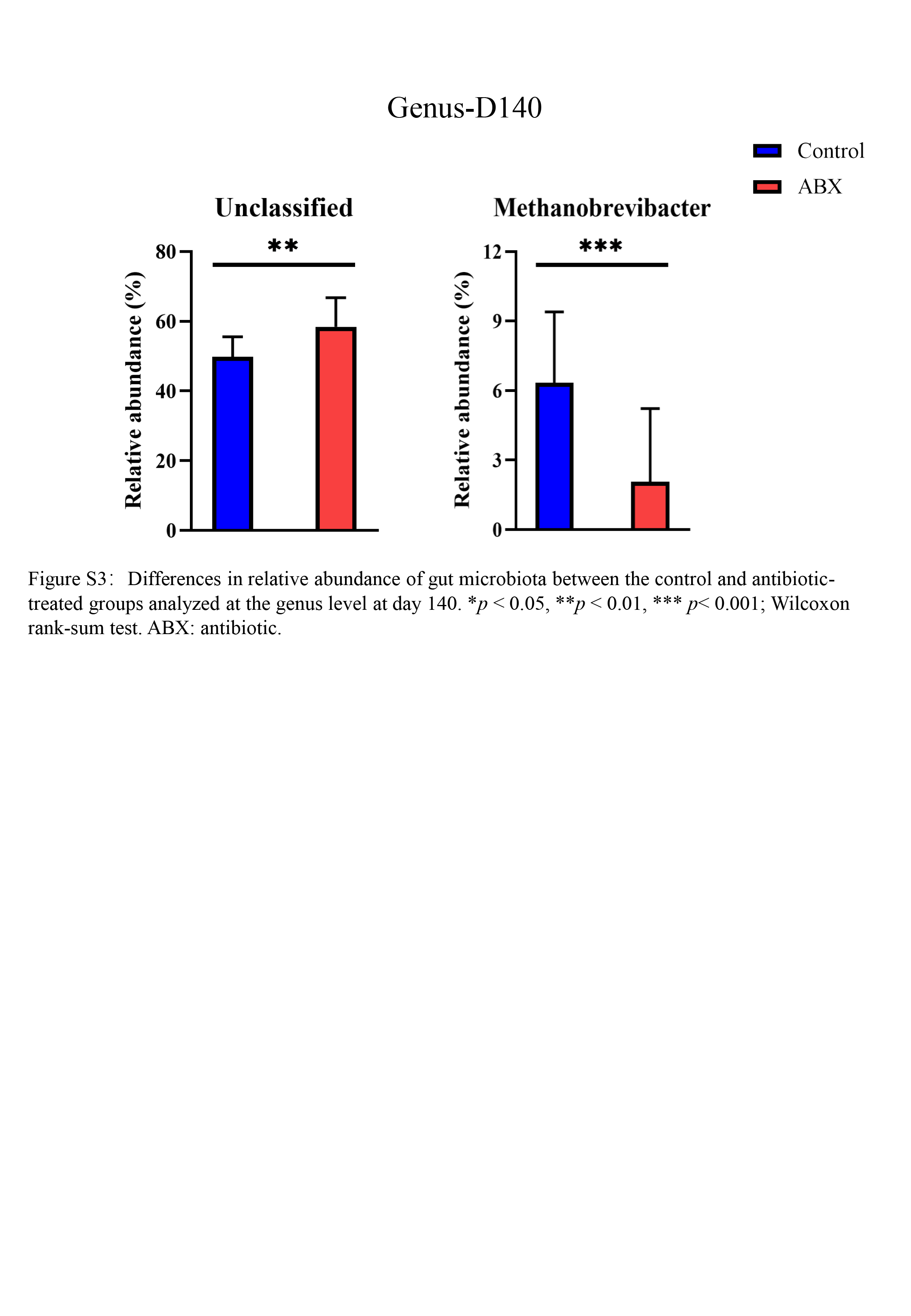

Supplement: Supplementary file 3 [file Image_3.tif]
